# Changes in smoking prevalence and cessation support, and factors associated with successful smoking cessation in Swedish patients with asthma and COPD

**DOI:** 10.1080/20018525.2017.1421389

**Published:** 2018-01-04

**Authors:** Marcus Stegberg, Mikael Hasselgren, Scott Montgomery, Karin Lisspers, Björn Ställberg, Christer Janson, Josefin Sundh

**Affiliations:** ^a^ School of Medical Sciences, Örebro University, Örebro, Sweden; ^b^ Clinical Epidemiology and Biostatistics, Örebro University, Örebro, Sweden; ^c^ Clinical Epidemiology Unit, Department of Medicine, Karolinska Institutet, Stockholm, Sweden; ^d^ Department of Epidemiology and Public Health, University College, London, UK; ^e^ Department of Public Health and Caring Sciences, Family Medicine and Preventive medicine, Uppsala University, Uppsala, Sweden; ^f^ Department of Medical Sciences, Respiratory, Allergy & Sleep Research, Uppsala University, Uppsala, Sweden; ^g^ Department of Respiratory Medicine, School of Medical Sciences, Örebro University, Örebro, Sweden

**Keywords:** Smoking prevalence, smoking cessation support, primary care, secondary care, cardiovascular risk factors, high educational level

## Abstract

**Introduction**: Our aim was to investigate changes in smoking prevalence, smoking cessation support and factors associated with successful smoking cessation in patients with asthma and COPD.

**Methods**: Questionnaires about available smoking cessation resources were completed by 54 primary health-care centers and 14 hospitals in central Sweden in 2005 and 2012. Patient data were collected using record reviews and patients questionnaires for two cohorts of randomly selected asthma and COPD patients in 2005 (*n* = 2306; with a follow up in 2012), and in 2014/2015 (*n* = 2620). Smoking prevalence, available individual and group smoking cessation support, and factors associated with successful smoking cessation were explored.

**Results**: Smoking prevalence decreased from 11% to 6% (*p* < 0.0001) in patients with asthma but was almost unchanged in patients with COPD (28 to 26%, *p* = 0.37). Smoking cessation support increased from 53% to 74% (*p* = 0.01). A high cardiovascular risk factor level, including diabetes mellitus and hypertension was associated with improved smoking cessation in patients with asthma (OR (95% CI) 3.87 (1.04–14.4), *p* = 0.04). A higher magnitude success was observed in men with asthma (OR (95% CI) 27.9 (1.73–449), *p* = 0.02). More highly educated women with asthma had successful greater smoking cessation (4.76 (1.22–18.7), *p* = 0.04). No significant associations were found in COPD.

**Conclusions**: The smoking prevalence in patients with asthma but not in COPD has almost halved in Sweden during a 7-year period. The availability of smoking cessation support has increased. Suggested factors related to successful smoking cessation are higher level of education in women with asthma and cardiovascular risk factors in men and women with asthma.

## Introduction

Tobacco smoking is an important risk factor for several diseases, such as chronic obstructive pulmonary disease (COPD), lung cancer, other forms of malignancies and cardiovascular diseases [1]. Tobacco smoking is also associated with a worse prognosis in asthma, as well as in COPD [2,3].

The mean prevalence of tobacco smoking in adults in Europe was estimated as 28% in 2015 [4]. In Sweden, a decreasing trend has been seen in both men and women over recent decades, and was estimated as 9%, in 2016 [5]. The custom of using moist snuff (‘snus’) in Sweden has been suggested as one of the explanations for the lower smoking prevalence [6,7]. According to the Swedish national register of asthma and COPD in 2016, 40% of patients with COPD were smokers. The proportion of smokers in patients diagnosed with asthma was 12% [8].

There are several interventions used for smoking cessation, including pharmacological therapy and counseling. Pharmacological options are nicotine replacement therapy (NRT), varenicline and bupropion, and counseling includes motivational interviewing and behavioral support performed as individual or group therapy. In a recent meta-analysis the combination of counseling and pharmacological therapy was most successful for smoking cessation compared with a minimal intervention or usual care [9]. According to the international recommendations Global initiative on Obstructive Lung Disease (GOLD) and Global Initiative for Asthma (GINA), smoking cessation is of great importance. Subsequently, smoking cessation has acquired the highest priority level in the Swedish National Board of Health and Welfare guidelines for asthma and COPD care [10].

Factors associated with successful smoking cessation in the general population are older age, male sex and higher educational level [11,12]. In COPD, a lower frequency of cessation has been found in patients who are younger, or have lower income, milder disease, high nicotine dependence and low self-efficacy [13–15]. However, there are still limited data on factors associated with successful smoking cessation in patients with asthma and COPD. The aim of this study was therefore to investigate whether the prevalence of smoking in asthma and COPD patients and availability of smoking cessation support have changed over a 10-year period in Sweden, and to identify factors associated with successful smoking cessation in patients with asthma and COPD.

## Material and methods

### Data collection

**Figure 1.**
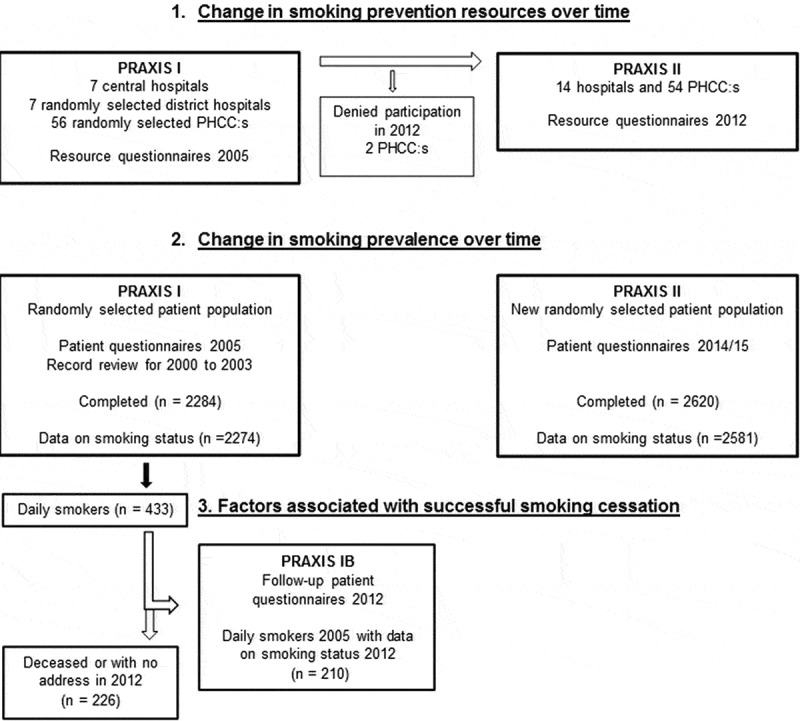
Flow chart. Flow chart of cohorts and data collection for the three major research questions of the study. PRAXIS I is referring to the first cohort from 2005, PRAXIS IB to the follow-up in 2012 of the first cohort, and PRAXIS II to the second cohort from 2014/2015. PHCCs = primary health care centers.

Patient data were collected from the PRAXIS study, an observational study involving seven county councils in central Sweden [16–26]. In the first PRAXIS-cohort, each county council was represented by the department of respiratory medicine in their central hospital, the department of internal medicine from one randomly selected district hospital and eight randomly selected primary health care centers (PHCCs). From the participating centers, a total of 3223 patients above 18 years of age with a doctor’s diagnosis of COPD (ICD-10, J44) or asthma (ICD-10, J45) were randomly selected. The first data collection was performed in 2005, using patient questionnaires and record reviews as well as resources questionnaires to the heads of the centers about available resources in asthma and COPD care. In 2012, follow-up questionnaires were sent to the patients from 2005 that were still alive. In 2012 new resources questionnaires were sent to the same centers, and new second cohorts were randomly selected in 2014 (COPD) and 2015 (asthma) from the same centers as in 2005. The data collection is summarized in the flow chart in Figure 1.

### Patient characteristics and measures

For comparison between the 2005 and 2014/15 cohorts, the patient questionnaires provided information on sex, age and smoking habits. Available smoking cessation resources (pharmacological treatment and behavioral support in terms of individual support, group support or both) and access to an asthma/COPD nurse at the participating centers were reported in the resources questionnaires from 2005 and 2012. For analyses of factors associated with smoking cessation between 2005 and the follow-up in 2012, information on sex, age, smoking habits, height, weight, educational level, exacerbations defined as number of emergency visits due to the obstructive disease in the last 6 months, and self-rated severity level of their disease was obtained from the 2005 patient questionnaire, and information about current smoking habits and received smoking cessation support from the follow-up questionnaire in 2012. In addition, data on the comorbid conditions hypertension, heart disease, diabetes and depression at baseline were collected in 2005 by review of records for the period 2000–2003.

Smoking history was categorized as current daily smoking or not. Body mass index was calculated as weight (kg)/height (m)^2^. Obesity was defined as body mass index (BMI) ≥30, overweight as BMI <30 and ≥25, normal weight as BMI <25 and ≥20 and underweight as BMI <20. The dichotomous educational variable identified the most highly educated group as 2 years or more beyond the Swedish compulsory schooling of 9 years. Exacerbations were grouped as 0, 1 or ≥2 during the previous 6 months. Self-estimated severity of disease included seven steps from mild to very severe disease. Heart disease was defined as having the diagnoses of ischemic heart disease or heart failure, and these, along with type 1 or type 2 diabetes were identified from records for the period 2000–2003. Depression was defined as having a diagnosis of depression in combination with antidepressant drug treatment during the period 2000–2003. A high cardiovascular risk factor level was defined as presence of both hypertension and diabetes diagnoses. Because of differences in the age distributions between patients with asthma or COPD, patients with asthma were categorized as ≤55, 56–65 and >65 years and patients with COPD were categorized as ≤65, 66–70 and >70 years.

### Statistical analysis

Analyses were performed using SPSS software version 23 (SPSS Inc., Chicago). Cross-tabulation and Pearson’s chi-square test were used to compare the smoking prevalence in the 2005 and 2014/15 cohorts, with stratification for sex and obstructive disease. McNemar’s test was used to investigate differences in available tobacco prevention support resources in the participating centers in 2005 compared with 2012. Logistic regression analysis with no daily smoking in 2012 as the dependent variable was performed among smokers from the 2005 population (*n* = 436), stratified by obstructive disease and sex. The independent variables were age (four groups), level of education, number of exacerbations (three groups), BMI (four categories), heart disease, depression, hypertension, diabetes, the variable for a high cardiovascular risk factor level, any smoking cessation support, access to asthma/COPD nurse and level of healthcare. Multivariable logistic regression analysis included statistically significant variables from the univariate analysis. Interaction analysis used interaction terms for sex with each relevant variable with adjustment for the main effects and the potential cofounding factors. An attrition analysis using the chi square test compared patients included in the regression analyses with patients smoking in 2005 but excluded due to missing data in 2012. A *p*-value below 0.05 was considered statistically significant.

### Ethical considerations

The study was approved by the Regional Ethical Review Board in Uppsala (Dnr:s 2004:M-445 and 2010/090). Written informed consent was given by all patients.

## Results

### Patient characteristics

Patient characteristics are shown in Table 1. In both cohorts, women were more common, and patients with asthma were generally younger and less often current daily smokers than the COPD patients.

**Table 1.** Patient characteristics.

	Asthma			COPD	
Characteristics	PRAXIS I *N* = 1195	PRAXIS II *N* = 1291	Characteristics	PRAXIS I *N* = 1089	PRAXIS II 1329
**Sex *n* (%)**			**Sex, *n* (%)**		
**Male**	475 (40)	498 (39)	**Male**	451 (41)	584 (44)
**Female**	720 (60)	793 (61)	**Female**	638 (59)	745 (56)
**Age**, *n* (%)			**Age**, *n* (%)		
**≤55**	685 (57)	605 (47)	**≤65**	549 (50)	416 (31)
**56–65**	294 (25)	279 (22)	**66–70**	281 (26)	408 (31)
**>65**	216 (18)	407 (31)	**>70**	259 (24)	505 (38)
**Age** mean (SD)	50 (15)	54 (16)	**Age** mean (SD)	64 (8)	61 (14)
**Smokers**, *n* (%)	125 (11)	74 (6)	**Smokers**, *n* (%)	308 (28)	345 (26)

Data presented as numbers and column percentages. PRAXIS I is referring to the first cohort in 2005, and PRAXIS II to the second cohort in 2014/2015. The alternative age intervals refer to the different age intervals in the asthma and respectively COPD cohorts. COPD: chronic obstructive pulmonary disease; SD: standard deviations; Smokers: current daily smokers.

### General smoking prevalence

**Figure 2.**
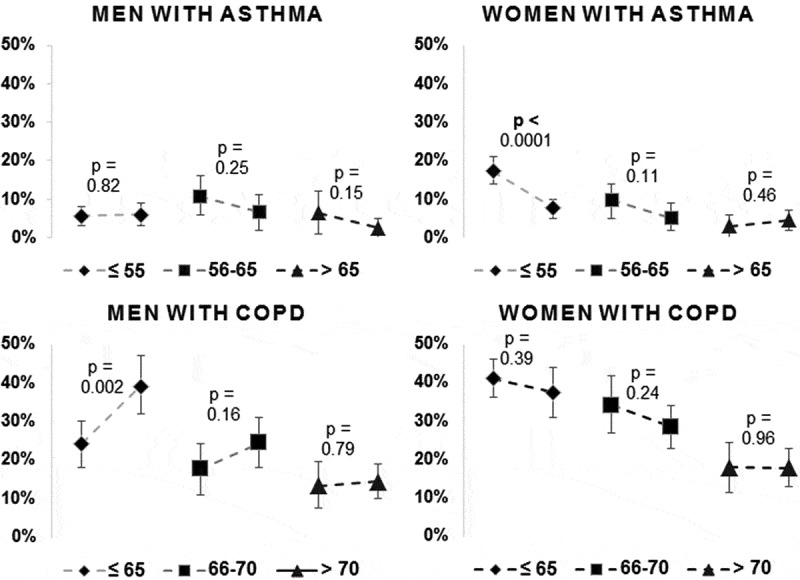
Smoking prevalence stratified by sex and obstructive disease. Prevalence of smoking in the two cohorts 2005 and 2014/15 stratified by sex and obstructive disease with 95% confidence interval. COPD: chronic obstructive pulmonary disease.

The prevalence of smoking in patients diagnosed with asthma decreased from 11% to 6% (*p* = <0.0001) between 2005 and 2015, whereas no statistically significant change was found in patients with COPD (28–26%, *p* = 0.37) between 2005 and 2014. In analyses with stratification for sex and obstructive disease, a statistically significant decrease in smoking prevalence was found in the youngest women diagnosed with asthma, and a statistically significant increase was shown in the youngest men diagnosed with COPD (Figure 2).

### Smoking cessation support resources

**Figure 3.**
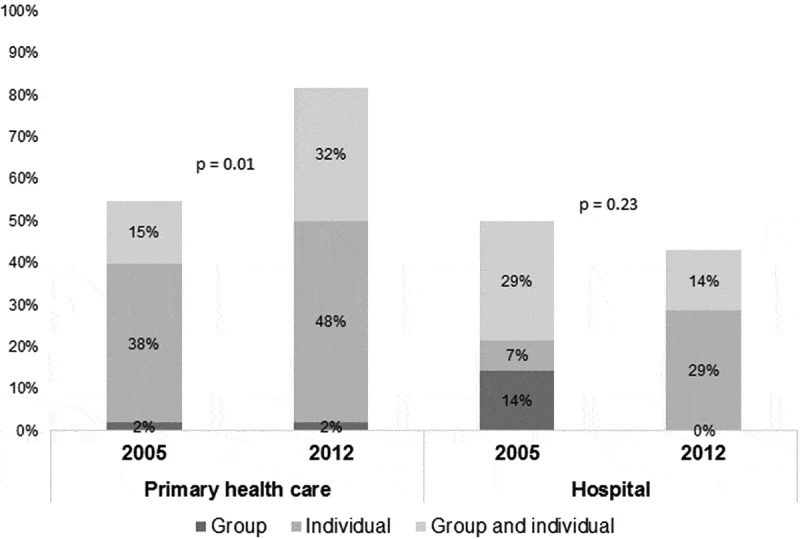
Smoking support. Available smoking cessation support in primary health care and in secondary care, 2005 vs. 2012.

The availability of smoking cessation support at the participating centers increased between 2005 and 2012 in the PHCCs, whereas no significant change was found in secondary care. However, in both primary and secondary care, the proportion of centers with individual therapy increased over time, and access to this type of support was more common than access to both individual and group support in 2012. Having only group therapy support was uncommon in primary care, and did not exist at all in hospitals in 2012 (Figure 3).

### Change of smoking habits and factors associated with quitting smoking

In total, 210 patients that were current daily smokers in 2005 were followed up in 2012. Of these, 112 (53%) remained smokers and 98 (47%) ceased smoking between 2005 and 2012. Most of the patients (62%) who ceased smoking between 2005 and 2012 used pharmacological therapy of some type. Only 27% ceased smoking with individual or group support from the health-care system. NRT was used by 29% and 22% had been prescribed bupropion or varenicline. Some 8% used both nicotine replacement therapy and bupropion or varenicline. In addition, 6% replaced smoking by using snuff. There were no statistically significant differences in using pharmacological smoking cessation support between patients with asthma and COPD.

In multivariable analysis, a high cardiovascular risk factor level was associated with successful smoking cessation in with asthma (OR (95% CI) 3.87 (1.04–14.4), *p* = 0.04). No significant associations were found in COPD. Results from multivariate logistic regression analysis with stratification for both sex and obstructive disease are shown in Table 2. The attrition analysis showed that among the patients who smoked in 2005, statistically significantly more with heart disease and aged over 65 years were lost to follow-up in 2012 (data not shown).

**Table 2.** Factors associated with successful smoking cessation.

	Men with asthma		Women with asthma		
Variables	Adjusted OR (95% CI)	*P*-value	Adjusted OR (95% CI)	*P*-value	*P* for interaction
High cardiovascular risk factor level	27.9 (1.73–450)	0.02	2.47 (0.48–12.8)	0.28	0.07
Higher level of education	0.33 (0.02–4.56)	0.41	4.76 (1.22–18.7)	0.03	0.04
High cardiovascular risk factor level	5.26 (0.94–29.6)	0.060	1.23 (0.49–3.08)	0.65	0.14
Higher level of education	0.49 (0.12–2.01)	0.32	0.78 (0.33–1.87)	0.58	0.53

Multivariable analysis stratified for sex and obstructive disease. The multivariable models included the explanation variables high cardiovascular risk factor level and higher educational level, as they were statistically significant in univariable analysis. OR: Odds Ratio; COPD: chronic obstructive pulmonary disease.

## Discussion

The main findings of this multicenter observational study were that the prevalence of smoking decreased in asthma but was unchanged in COPD patients during the last decade. The availability of smoking cessation support has increased in primary care between 2005 and 2012. A high cardiovascular risk factor level was associated with greater chance of smoking cessation in asthma.

### General smoking prevalence

The adverse effects of smoking in asthma and COPD are well known. Bronchial hyper responsiveness is a risk factor for developing COPD [27], and smoking cessation is of substantial importance in patients with asthma. In COPD, the disease progression in smokers compared with never smokers and ex-smokers is well established, and implies a benefit from stopping smoking regardless of the degree of lung function limitation [28].

The overall smoking prevalence in Sweden decreased from 14% to 11% between 2006 and 2012 [5]. In the seven county councils in Sweden represented in our study, the mean prevalence was between 12% and 16% over the 3 year period between 2004 and 2007 and from 9% to 12% over the period 2011–2014 [5]. Thus, current smoking prevalence is generally lower in patients with asthma and higher in patients with COPD compared with the general population.

Our study shows a lower tobacco smoking prevalence in patients with asthma and COPD compared with the report from the Swedish national register of asthma and COPD [8]. This could be because that the register is rather new and does not yet have sufficient coverage for all severity stages, and smoking patients may be prioritized for visits and thus for registrations. In addition, patients who ceased smoking during the previous 6 months are registered as current smokers. In our study, the random selection of patients corresponds to the distribution of severity stages of COPD in a large population-based Swedish study [29], and the patient questionnaires report the current smoking status.

We speculate that the decreased smoking prevalence in asthma but not in COPD could be because patients with COPD have a longer smoking history and a more severe nicotine dependence and so are less likely to be successful in smoking cessation. The current smokers in the baseline COPD cohort already represent a selected sample of patients who haven’t managed to stop smoking at the time of their diagnosis. Previous studies have shown that a lower nicotine dependence and high self-efficacy are associated with higher chance of smoking cessation [14,15].

The decrease in smoking prevalence in our study was greatest among women below age 55 years diagnosed with asthma, which might be an effect of the general decline in smoking prevalence among younger women in Sweden [5]. The increased smoking prevalence among men below age 65 years with COPD is interesting because it is not consistent with previous studies where patients with COPD were more likely to cease smoking compared with smokers in the general population [30,31]. The general prevalence of mild COPD at younger ages may have increased during the last decade due to higher awareness of the need to perform spirometry, and in this group of patients the rate of smoking cessation is lower [32].

### Availability of resources

In our study the majority of the centers in primary care but not in secondary care offered smoking cessation support. Both individual and group counseling have been shown to improve smoking cessation success [33]. Interestingly, the proportion of centers offering only individual therapy has increased in both primary and secondary care. The overall availability of smoking cessation support increased in primary care, and this may be due to implementation of national guidelines for both asthma and COPD and for methods of preventing disease from the Swedish National Board of Health and Welfare, resulting in a more developed organization for smoking support in primary care.

### Change of smoking habits and factors associated with smoking cessation

Previous studies have explored factors associated with successful smoking cessation attempts in the general population [11,12], and a study of more than 3000 hospital based patients with COPD has shown that older age, higher income and more severe disease are associated with a higher chance of stopping smoking. However, to our knowledge there are still limited data on factors associated with smoking cessation in patients with asthma, and in patients with obstructive disease managed in primary care. Our results show an association between having combined hypertension and diabetes mellitus with successful smoking cessation in asthma. There were also clear indications that hypertension and diabetes alone were factors of importance for smoking cessations, although most often not statistically significant due to small numbers. This may indicate the importance of improving the quality of discussion with patients about cardiovascular risk factors and the benefits of giving up smoking. We speculate that these patients have health care visits more regularly which may give more opportunities for brief intervention concerning smoking and thus influence their motivation to cease smoking.

The fact that no statistically significant associations were found for cardiovascular risk factors with smoking cessation in patients with COPD is of interest. However, the majority of the patients lost between 2005 and 2012 had COPD and most likely died from heart disease, and thus some patients with COPD who ceased smoking due to a high cardiovascular risk factor level may have been excluded. In addition, patients with asthma are generally younger, with lower amount of pack years and may be more receptive for the message about cardiovascular risk factors.

In women with asthma, a higher level of education was associated with greater smoking cessation, consistent with the results from previous studies of smokers in the general population [11]. This was not seen in men with asthma, possible due to lower numbers. The association with higher level education was not found in women or men with COPD or in men with asthma. Interaction analysis showed statistically significant effect modification by female sex on the association of higher level of education and smoking cessation in asthma.

### Strengths and limitations

The major strengths of our study include that it is a multicenter study of both primary and secondary care including both data on patient characteristics and on available resources, and with analysis of prevalence and factors associated with successful smoking cessation. The study was conducted using real world data ensuring a high external validity and generalizability.

A potentially major limitation is that although the original cohort included more than 2000 patients, a rather small proportion of the population were smokers. Some of the statistically significant results from the regression analysis had wide confidence intervals, due to low power. Thus, there may be more factors associated with smoking cessation, which we were unable to detect in this study due to a small number of patients, and due to missing data for smoking support. Between the 2005 cohort and the 2012 follow up there was a loss of patients. This was expected since the patients, mostly those diagnosed with COPD, have a higher mortality rate and more likely to die during follow up. Patients with more severe COPD are more likely to cease smoking because of their symptoms and are also more likely to die from their disease. In addition, most of the lost patients had heart disease which is the most common cause of death in COPD. Comorbid conditions may also have developed during the follow-up, and thus not included as independent factors at baseline. We find it interesting that a high cardiovascular risk factor level although not heart disease was associated with successful smoking cessation in COPD, and speculate that this may be because people with cardiovascular risk factors but no existing heart disease may be more motivated to stop smoking. Another limitation is that we have no data on the patients’ motivation or self-efficacy for smoking cessation. Finally, patients in this study were included based on a doctor’s diagnosis of COPD or asthma, which may be incorrectly recorded and thereby influence the results in our study.

### Clinical implications and need of further research

The results in this study are of importance to patients who smoke and are diagnosed with asthma or COPD and managed in either primary health care or hospitals. We also believe it is of substantial benefit to clarify to patients the increased risk of tobacco use when other cardiovascular risk factors are present. Future studies might investigate the importance of frequent health care visits in this context. A comparison of the impact of different types of smoking cessation support would also be of great interest, and should be performed in a study with more complete data on these factors. Finally, an important implication is that better smoking cessation support is needed, especially in hospitals.

## Conclusion

The general prevalence of smoking in patients with asthma but not in COPD has decreased over the last decade and resources for smoking support increased between 2005 and 2012. Overall and specifically in men with asthma, smoking cessation seems to be more common in patients with a higher cardiovascular risk factor level with diabetes and hypertension. In women with asthma smoking cessation is more common among those with a higher level of education.

## Geolocation information

The study was performed in central Sweden.
